# Adaptive Evolution of CENP-A in Percid Fishes

**DOI:** 10.3390/genes6030662

**Published:** 2015-07-17

**Authors:** Harriet N. A. Abbey, Leos G. Kral

**Affiliations:** Department of Biology, University of West Georgia, Carrollton, GA 30118, USA; E-Mail: hallietus@gmail.com

**Keywords:** CENP-A, adaptive evolution, percids, fishes

## Abstract

Centromeric protein A (CENP-A) is the epigenetic determinant of centromeres. This protein has been shown to be adaptively evolving in a number of animal and plant species. In a previous communication we were able to demonstrate that signs of adaptive evolution were detected in the comparison of CENP-A sequences from three percid fish species. In this study we isolated the CENP-A gene from eight additional species from the Percidae family. With these sequences and those previously obtained, we carried out a more robust statistical analysis of codon specific positive selection in CENP-A coding sequences of eleven percid species. We were able to demonstrate that at least two amino acid positions within the N-terminal tail are under strong positive selection and that one of these positions is potentially a substrate for phosphorylation. While nonsynonymous substitutions were detected in the histone fold domain, these were not statistically supported as resulting from positive selection.

## 1. Introduction

Centromeres appear to be epigenetically determined by the deposition of centromeric protein A (CENP-A) which is a histone H3 variant that replaces histone H3 in nucleosomes that form the basis of kinetochore assembly (see reviews [1,2]). Comparative analysis of CENP-A in related groups of species has shown that CENP-A undergoes adaptive evolution. This adaptive variation was shown to occur in the N-terminal tail and a portion of the histone fold domain (HFD) of CENP-A in primates [3], the *Drosophila* ortholog Cid [4], and the Brassicaceae family ortholog CenH3 [5,6]. Adaptive variation has been hypothesized to be due to female meiotic drive where those centromeres that are able to attract a greater number of centromeric proteins will form a stronger kinetochore and thus be selected by preferential segregation into the egg. Imbalance of centromere strengths would lead to nondisjunction during spermatogenesis where centromeric proteins that suppress formation of the strong centromeres would be selected [7]. It has been shown in mice that female meiotic drive is indeed the result of strong centromeres that attract a greater number of centromeric proteins [8].

**Figure 1:**
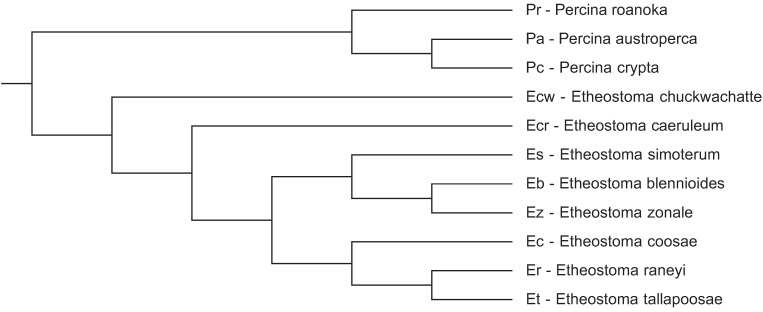
Cladogram of the evolutionary relationships of darter species utilized in this study. The darter species tree from Near *et al*. [17] was pruned to reflect the topology of these 11 species.

In a previous communication [9] we compared CENP-A from three fish species in the percidae family and obtained a positive selection signal in the N-terminal tail. In this communication we present data from an expanded number of darter species in this family to permit a more robust statistical analysis of codon specific positive selection in CENP-A. Species were selected to provide a range of close and distant evolutionary relationships (Figure 1) to assess if CENP-A variation could, potentially, lead to speciation in darters.

## 2. Experimental Section

### 2.1. DNA Source Materials and DNA Isolation

*Etheostoma chuckawachatte* was locally obtained form Walker Creek in Haralson County, GA and *Percina crypta* was locally obtained from Yellowdirt Creek in Carroll County, GA. *Etheostoma coosae* was obtained from Dr. Mark Meade, Jacksonville State University, Jacksonville, Alabama. *Etheostoma raneyi* was obtained from Dr. Mel Warren, USDA Forest Service, Oxford, Missouri. *Etheostoma blennioides*, *Etheostoma caeruleum*, *Etheostoma simoterum* and *Etheostoma zonale* were obtained from Dr. Bruce Stallsmith, University of Alabama in Huntsville, Huntsville, Alabama. DNA for sequencing was obtained from one specimen of each of these species.

Genomic DNA was isolated from fin and, perhaps, some adjoining muscle tissue samples with either the Qiagen DNeasy Blood and Tissue Kit according to manufacturer’s instructions (including optional RNase treatment step) or with the Quick-gDNA MiniPrep kit from Zymo Research (Irvine, CA, USA) utilizing the manufacturer’s solid tissue samples protocol where the fin tissue was mechanically homogenized in Genomic Lysis Buffer in 1.5 mL microcentrifuge tubes. The genomic DNA was then used for either PCR amplification or whole genome sequencing.

### 2.2. PCR Amplifications of Darter CENP-A Genes

The entire CENP-A gene was PCR amplified from *E. blenoides*, *E. coosae*, *E. caeruleum*, *E. simoterum* and *E. zonale* genomic DNA utilizing primers EAFi and revMTF6 which are complementary to conserved exon 1 sequences of the two genes directly adjacent to the CENP-A gene in darters [9]. The PCR products ranged from 5700 to 8000 base pairs. The reaction mixture was composed of 25 μL of RANGER mix polymerase (Bioline), 320 ng of genomic DNA, and 0.5 μM of each primer in a total volume of 50 μL. The PCR conditions were 95 °C for 5 min to activate the DNA polymerase followed by 35 cycles of 20 s at 98 °C, 45 s annealing at 63 °C and 8 min at 66 °C. The final cycle was followed by a 10 minute incubation at 72 °C.

Possibly due to the somewhat degraded nature of the genomic DNA isolated from *E. raneyi*, the CENP-A gene had to be PCR amplified in two parts. The part of the CENP-A gene containing exon 1, exon 2, exon 3, and a portion of exon 4 were amplified with forward primer EAFi and reverse primer tdA3S which is complementary to an *Etheostoma tallapoosae* exon 4 sequence of the four exon long CENP-A gene [9]. Once the sequence of this 6100 base pair long amplimer was determined, the intron sequence upstream of the exon 4 sequence was examined. It was found that a previously designed primer fwPA1 [9] was complementary to a portion of this intron sequence. PCR amplification of exon 4 of the CENP-A gene was carried out with this fwPA1 primer and the revMTF6 primer and yielded a 2000 base pair long amplimer. These PCR reactions were carried out as detailed above.

### 2.3. DNA Sequencing

All CENP-A gene amplimers were gel purified utilizing the Zymo Gel DNA Recovery Kit (Zymo Research). Where necessary, purified amplimers from multiple PCR reactions of the same species were combined and concentrated using the DNA Clean and Concentrator Kit (Zymo Research) to obtain the required 1 µg of DNA at 50 ng/µL.

Separately fragmented and barcoded amplimers of the entire CENP-A gene from *E. blenoides*, *E. coosae*, *E. caerulum*, *E. simoterum*, and *E. zonale* and of the *E. raneyi* amplimer spanning exons 1, 2, 3, and 4 (partial) were sequenced by the Georgia Genomic Facility at the University of Georgia utilizing the Illumina MiSeq sequencer. Paired end reads of 250 bp were obtained from each species in sufficient quantity to yield about a 10,000 fold coverage of each of the CENP-A genes.

Sanger sequencing of the *E. raneyi* exon 4 containing amplimer was carried out by Functional Biosciences, Inc., Madison WI (functionalbio.com).

Whole genome sequencing was performed on *E. chuckawachatte* and *P. crypta* genomic DNA by the Georgia Genomic Facility at the University of Georgia utilizing the Illumina NextSeq sequencer. Paired end reads of 150 bp were obtained from each species in sufficient quantity to yield more than 30 fold coverage of the CENP-A gene.

### 2.4. DNA Sequence Assembly

A subset of amplimer PE 250 sequences from each species sufficient for at least 30 fold coverage was aligned to the *E. tallapoosae* CENP-A gene reference sequence [9] utilizing the Map to Reference function in Geneious software (Biomatters Ltd., Auckland, New Zealand). The whole genome *E. chuckawachatte* and *P. crypta* PE 150 sequences were also aligned to the *E. tallapoosae* CENP-A gene reference sequence by the same method. The respective consensus sequences of each of the four exons were then concatenated to assemble the CENP-A coding sequence for each of the species. The assembly of the initial *E. raneyi* amplimer only yielded a partial coding sequence spanning exons 1, 2, 3, and 4 (partial). The entire *E. raneyi* CENP-A coding sequence was then reconstructed by incorporating the Sanger sequence of the exon 4 amplimer. All darter CENP-A gene sequences used in this study are available in GenBank under the following accession numbers: HQ203081, KR610394, KR610395, KR610396, KR610397, KR697767, KR697768, KR697769, KR697770, KR697771, KR697772.

### 2.5. Testing for Positive Selection and Potential Posttranslational Modifications

All of the darter CENP-A coding DNA sequences obtained in this study as well as those of *E. tallapoosae*, *Percina austroperca,* and *Percina roanoka* obtained previously [9] were aligned and subject to site specific tests for positive selection utilizing the Selecton server implementation of an empirical Bayes approach [10,11] and also the HyPhy package on the Datamonkey webserver [12] (www.datamonkey.org) consisting of the following codon-based maximum likelihood methods: The Single Likelihood Ancestor Counting model (SLAC), the Fixed Effect Likelihood model (FEL), the Random Effect Likelihood model (REL), the Mixed Effects Model of Evolution (MEME) and the Fast Unbiased Bayesian Approximation (FUBAR). Prior to these analyses, the best fitting model of nucleotide substitution was determined with the model selection tool available on the Datamonkey server. Possible sites of phosphorylation were determined with the NetPhos 2.0 server [13] (http://www.cbs.dtu.dk/services/NetPhos/) as well as with the DISPHOS 1.3 server (http://www.dabi.temple.edu/disphos/).

## 3. Results and Discussion

**Figure 2:**
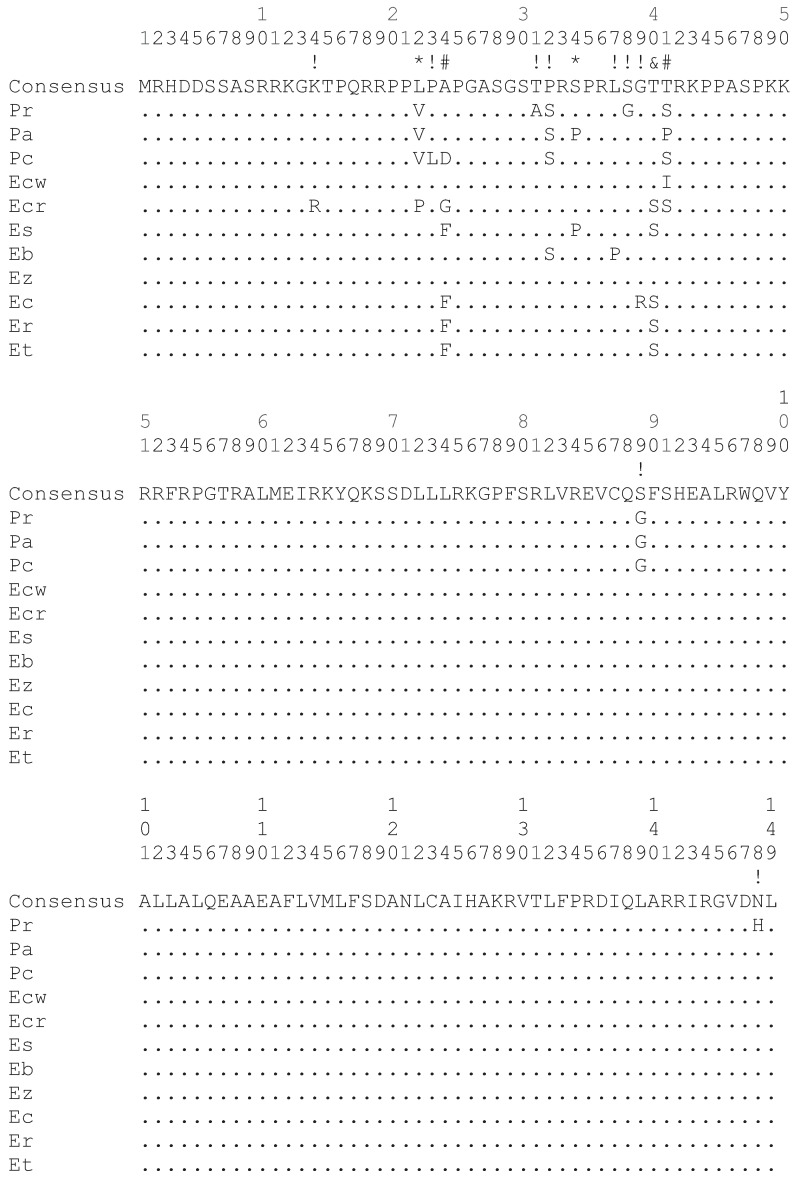
Alignment of the deduced CENP-A protein sequence from all 11 species of darters. Differences from the consensus sequence are indicated by single letters and dots indicate amino acids identical to the consensus. The N-terminal tail spans amino acids 1 to 56. The HFD α1 helix spans amino acids 76 to 90 and the HFD loop 1 region spans amino acids 91 to 98. Symbols above the consensus sequence indicate K_A_/K_S_ ratio is greater than 1 where (*) indicates that these were found to be significant by REL only, (#) indicates that these were found to be significant by all methods except SLAC, (&) indicates that these were found to be significant by Selecton and REL, and (!) indicates that these were not statistically significant. Pr: *Percina roanoka*, Pa: *Percina austroperca*, Pc: *Percina crypta*, Ecw: *Etheostoma chuckawachatte*, Ecr: *Etheostoma caerulum*, Ec: *Etheostoma coosae*, Et: *Etheostoma tallapoosae*, Er: *Etheostoma raneyi*, Es: *Etheostoma simoterum*, Ez: *Etheostoma zonale*, Eb: *Etheostoma blennioides*.

The darter CENP-A coding sequences determined in this study as well as those determined in a previous study [9] were aligned and subject to a number of different analyses for positive selection at individual codon sites utilizing the ratio of non-synonymous substitution rates (K_A_) in relation to synonymous substitution rates (K_S_). Positive selection is indicated if the K_A_/K_S_ ratio is greater than 1. As shown in Figure 2, most of the non-synonymous substitution sites are present in the N-terminal tail of the darter CENP-A protein coding sequences. One non-synonymous substitution site is present in the α1 helix of the HFD and one non-synonymous site is present in the C-terminal tail. While the loop 1 region of the *Drosophila* and Brassicaceae CENP-A orthologs has been shown to be under positive selection [4,6], no non-synonymous substitutions occur in this site in the darter sequences. The only statistical support obtained for positive selection in the darter CENP-A was for sites within the N-terminal tail (Figure 2).

Of the various methodologies employed, all provide statistical support for positive selection of sites within the N-terminal tail (Figure 2) except SLAC. The SLAC method is the most conservative of the methods utilized [14]. The empirical Bayes approach implemented on the Selecton server identified twelve sites at which the K_A_/K_S_ ratio is greater than 1 (Figure 2). Of these, three sites at positions 24, 40 and 41 were deemed to reliably support positive selection since the lower bounds of the confidence intervals of the Bayesian estimates of K_A_/K_S_ ratio is greater than 1. The Selecton server implementation also performs a significance test by calculating a likelihood ratio test between the M8 model which allows positive selection [15] and the M8a model which does not [16]. This likelihood ratio test between the two models shows a significance level of 0.01 when a neighbor joining tree is calculated by default by the server software and a significance level of 0.001 when a phylogenetic tree corresponding to the evolutionary relationships between the darter species determined by Near *et al*. [17] is provided instead, thus indicating that the CENP-A gene does undergo positive selection in the darters.

The FEL, REL, MEME, and FUBAR methods implemented in the HyPhy package also supported sites 24 and 41 as being under positive selection utilizing the default significance levels implemented on the Datamonkey server. The REL method also supported site 40 as being under positive selection in agreement with the Selecton server results and also identified sites 22 and 34 as two additional sites under positive selection. The REL method, however, is susceptible to falsely identifying positive selection sites in small datasets [14].

**Figure 3:**
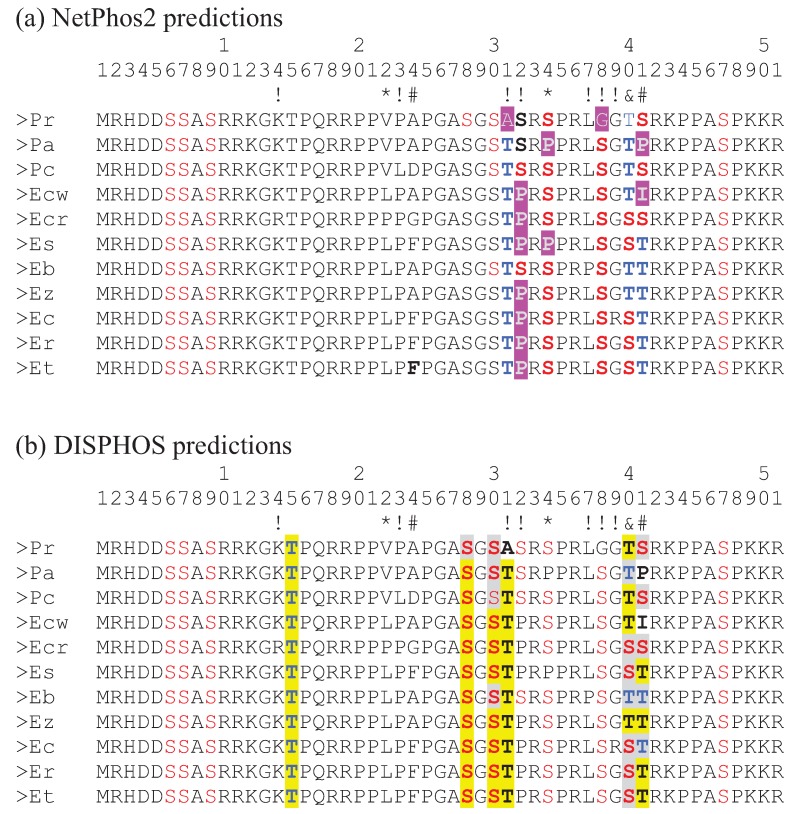
Alignment of the N-terminal tail amino acid sequences form the 11 darter species. Symbols below the numbers indicate significance of K_A_/K_S_ ratio greater than 1 as detailed in Figure 2. (**a**) Phosphorylation sites predicted by NetPhos2 are colored red (serine) and blue (threonine). Amino acids highlighted in purple indicate changes to amino acids that do not serve as phosphorylation substrates. (**b**) Phosphorylation sites predicted by DISPHOS are colored red (serine) and blue (threonine). Differences of S/T phosphorylation predictions by NetPhos2 and DISPHOS are highlighted in yellow. Identical predictions by the two methods are highlighted in gray. Pr: *Percina roanoka*, Pa: *Percina austroperca*, Pc: *Percina crypta*, Ecw: *Etheostoma chuckawachatte*, Ecr: *Etheostoma caerulum*, Ec: *Etheostoma coosae*, Et: *Etheostoma tallapoosae*, Er: *Etheostoma raneyi*, Es: *Etheostoma simoterum*, Ez: *Etheostoma zonale*, Eb: *Etheostoma blennioides*.

The N-terminal tail of CENP-A is known to be posttranslationally modified in *S. cerevisae* [18] as well as in human cells [19,20,21]. Specifically, the number 7 serine has been known to be phosphorylated in human CENP-A protein and the phosphorylation is necessary for the mitotic function of centromeres [20]. In their analysis of adaptive variation of CENP-A in primates, Schueler *et al*. [3] identified site 17 as being under positive selection. This site was subsequently identified as another serine phosphorylation site in human CENP-A which, along with phosphorylation site 19, is also necessary for mitotic function of centromeres [21]. Note that in the Bailey et al. study [21] these two serine sites are actually numbered 16 and 18 since their numbering scheme excludes the initiating methionine that is cleaved off after translation. Interestingly, site 41 in the darter CENP-A, identified by consensus of the methods used as being under positive selection, is predicted as being a serine/threonine phosphorylation site in a number of the darter species examined (Figure 3). In *P. austroperca* (Pa) and *E. chuckawachatte* (Ecw), amino acids are substituted at this position which do not serve as phosphorylation substrates. In some of the darter species position 41 is occupied by a serine while in some of the other darter species threonine is present. While both of these amino acids serve as substrates for serine/threonine kinases, the efficiencies of phosphorylation of serine and threonine may be different and, indeed, the predictions of threonine phosphorylation at this position generated by NetPhos 2 and by DISPHOS are not in agreement for several of the species. Similarly, site 40, which has been identified as being under positive selection by the Selecton server method and by the REL method is occupied by serine in some species and threonine in the other species and this site is also predicted to be a phosphorylation site. However, the disagreement about threonine phosphorylation by NetPhos 2 and by DISPHOS again raises the possibility that these substitutions may not be functionally interchangeable.

It is also interesting that sites 31, 32, 34, and 38, predicted to be phosphorylation substrates by at least one of the prediction methods, are also sites where non-synonymous substitutions result in the replacement of a serine or a threonine with an amino acid that is not a substrate for kinases. These substitution sites are not, however, statistically supported as sites of positive selection except site 34 which is statistically supported by REL.

It has been shown that the N-terminal tail of CENP-A does appear to have a specific function during meiosis that is distinctly different than its function in mitosis. This functional difference was revealed in studies where various N-terminal GFP tagged chimeric CENP-A constructs were tested for their ability to rescue CENP-A null mutants in *A. thaliana* [22,23]. Specifically, N-terminal GFP tagged chimeras comprised of the native *A. thaliana* CENP-A HFD and either *A. thaliana* histone 3.3 N-terminal tail or *Z. mays* CENP-A N-terminal tail were able to rescue the lethal CENP-A null phenotype of the respective transformants but these transformants were sterile [22,23]. That is, these N-terminal GFP tagged chimeras were able to localize to mitotic centromeres and support mitosis but were depleted from meiotic centromeres thus disrupting kinetochore assembly and meiotic segregation of chromosomes [23]. Transformants of null mutants with native N-terminal GFP tagged CENP-A were both viable and fertile [23]. We are not aware of any studies of CENP-A phosphorylation during meiosis but it is possible that this protein could be subject to meiosis specific phosphorylation. Therefore, some of the adaptive evolution of the N-terminal tail could, potentially, lead to species specific meiotic phosphorylation patterns which could, at least in part, be responsible for the distinct meiotic function of this portion of CENP-A.

If positively selected variation in the CENP-A N-terminal tail is functionally related to meiosis, it is likely that meiotic incompatibility may result in hybrids that express CENP-As with divergent N-terminal tails. As such, adaptive variation in the N-terminal tail may either lead to speciation or may reinforce species. Some of the sympatric darter species, for example *E. caerulum* (Ecr) and *E. simoterum* (Es), differ in as many as five non-synonymous substitutions in their N-terminal tails (Figure 2) and thus, potentially, these differences could provide species reinforcement. However, there is, as yet, no data to indicate how many and what type of changes could give rise to hybrid incompatibility. It is likely, though, that the rate at which adaptive variation in the N-terminal tail arises is not fast enough to directly lead to speciation in darters. Two very closely related allopatric species, *E. tallapoosae* (Et) and *E. raneyi* (Er) have identical N-terminal tail sequences and a closely related species *E. coosae* (Ec) sharing a common ancestor with both of these differs by only a single amino acid (Figure 2).

It would be interesting to examine viable hybrids between various darter species and determine if any correlation exists between the sequence divergence of the N-terminal tails and the degree of infertility due to meiotic errors. It would also be interesting to examine the structure of centromeric DNA and sequence variation of other centromeric proteins in darter species to determine if these co-evolve with CENP-A. At this time there have been no studies published on these topics in darters, but we are in the process of examining other centromeric proteins in a subset of distantly related darter species for signs of adaptive evolution.

## 4. Conclusions

CENP-A sequences were obtained from a number of darter species that were sufficiently diverged to allow for analysis of adaptive evolution. Tests of codon specific selection showed that at least two amino acid positions in the N-terminal tail of CENP-A undergo positive selection and that one of these positions is a potential phosphorylation site.
